# Supplementary material for: P-TRAP: a Panicle Trait Phenotyping tool
Source: BMC Plant Biol. 2013 Aug 29;13:122. doi: 10.1186/1471-2229-13-122 (PMC3848748; doi:10.1186/1471-2229-13-122)
Supplement: Additional file 7 — P-TRAP source code. The Netbeans project that contains the source code of the application. [file 1471-2229-13-122-S7.zip › javadoc.zip!/javadoc/org-bioalg-skeletonprocessor/allclasses-frame.html]

All Classes (SkeletonProcessor)


# All Classes

- GraphProducer
- Skeleton
- Skeleton.PIXEL\_TYPE
- SkeletonExteractor
- SkeletonProcessor
